# Flipping the flipped: the co-creational classroom

**DOI:** 10.1186/s41039-018-0077-9

**Published:** 2018-07-31

**Authors:** Vuk Uskoković

**Affiliations:** 1 Department of Biomedical and Pharmaceutical Sciences, Chapman University, 9401 Jeronimo Road, Irvine, CA 92618-1908 USA; 2 Department of Bioengineering, University of Illinois, 851 South Morgan Street, Chicago, IL 60607-7052 USA

**Keywords:** Active learning, Bioengineering, Bloom taxonomy, Co-creation, Constructivism, Didactics, Flip, Flipped Learning Network, Medical devices, Natural science, Technology

## Abstract

The flip teaching model is being increasingly adopted by higher education institutions as an active learning alternative to traditional lecturing. However, the flip model shares a number of critical premises with the classical didactics. The further flips of the flip are thus advocated and the fear of returning the method to its initial state, prior to the flip, via such flips of the flipped dispelled. Proposed here is a seminal variation to the flip model based on the active involvement of students in searching, finding, selecting, and assembling knowledge from various literature sources into the learning material for the entire class. Because students actively co-create the learning content together with other students and the instructor, one such open-ended collaborative model is christened “co-creational.” Its conception and corollaries in relation to co-educational methods in general are discussed. The model is represented algorithmically, exemplified by a topic of choice and compared in a quasi-experimental setting against the standard flip and the traditional lecturing in a medical devices graduate class. Students were able to retain and reproduce the content covered using the co-creational pedagogic method better than using the standard flip or traditional lecturing. They also had a positive perception of the method, as compared to traditional lecturing. They did not have a preference for the co-creational method over the standard flip, but felt that they learned more using the co-creational method compared to the standard flip and that the co-creational model best prepared them for job searches in high-tech industry and academia. The co-creational model was also more open to the intrusion of moral instructions than traditional lecturing, going hand-in-hand with the community-building aspect of the ideal form of knowledge acquisition and creation.

**Electronic supplementary material:**

The online version of this article (10.1186/s41039-018-0077-9) contains supplementary material, which is available to authorized users.

## Introduction

### The flip

“There must be an ongoing recognition that everyone influences the classroom dynamic, that everyone contributes. These contributions are resources. Used constructively they enhance the capacity of any class to create an open learning community. Often before this process can begin there has to be some deconstruction of the traditional notion that only the professor is responsible for classroom dynamics”

Bell Hooks, 1994.

**Fig. 1:**
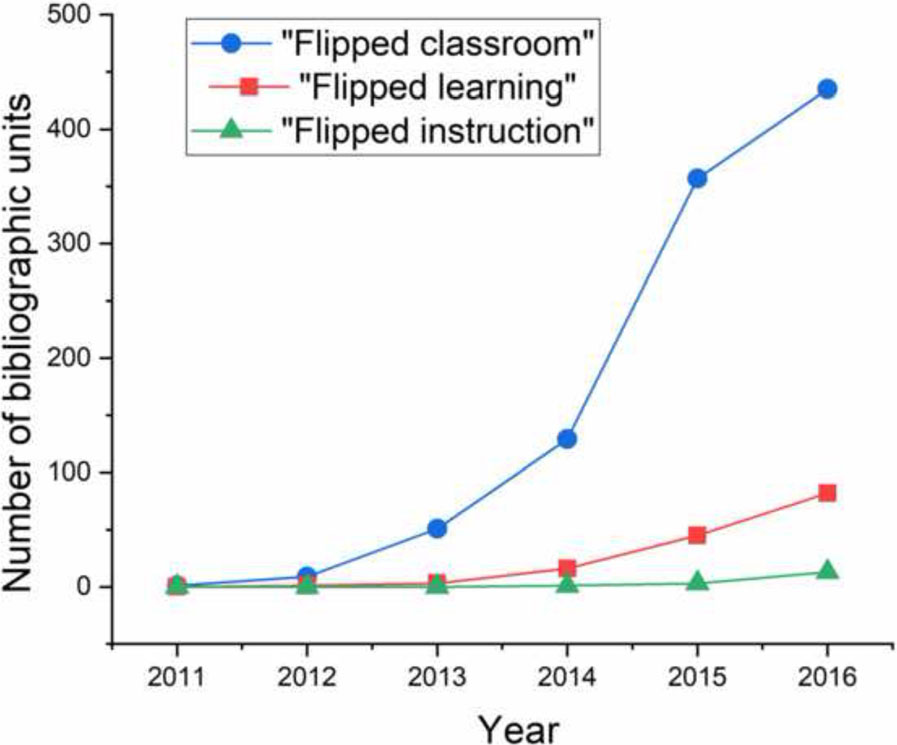
The literature trend. Annually published number of scholarly, peer-reviewed journal articles containing the phrases “flipped classroom,” “flipped learning,” or “flipped instruction” in the 2011–2016 period (Database: Web of Science Core Collection, December 2017)

Much has been said about the flipped classroom, an active learning model that enters the college and university classrooms at a faster pace than any other active learning model (Bergmann and Sams 2015). Albeit radical, the idea of the flip emerged gradually from the broader scope of active learning efforts. It came out as a natural corollary of gradually decentralizing the classroom through these efforts and distributing the power of spoken word across the class as a whole. Although various variants of this method have been practiced since antiquity (Sams and Bergmann 2013), the flip as a term did not emerge before the turn of the twentieth century. The first mention of inverted classroom, a term synonymous with the flipped classroom, dates back to year 2000 and an attempt to implement this new model in a higher education economics course (Lage et al. 2000), soon after which the phrase “classroom flip” was first reported (Baker 2000). The term remained in an embryonic stage for the next decade before its terminological kin, “flipped classroom,” became coined (Sams 2011) and educators got accustomed to it, resulting in an explosively fast adoption of the phrase since 2011. Since natural science education has been more focused on explicit problem-solving, the idea of flipping the classroom emerged from the academic domain where it seemed most revolutionary: humanities. Colloquially, the flipped instructional strategy is also often associated with technological aids that facilitate learning and mediate communication (LaFee 2013). Because of this informal reliance on high-tech tools, the flip was initially most enthusiastically embraced and innovated upon by educators in comparatively affluent, private higher education settings before its wave swamped all other types of academic institutions, from public preschools (PRWeb Newswire 2013) to elementary (Aidinopoulou and Sampson 2017), middle (Sezer 2017), and high (Leo and Puzio 2016) schools to the world’s most prestigious research universities (Moore et al. 2014). The expansion of this instructional method in the applicative domain has been paralleled by the intensification of research on it, and as a result, the number of peer-reviewed reports on “flipped classroom” has grown continuously since its seminal report in the summer of 2011 (Fig. 1).

The premises of the flip are simple. Contrary to what the notion of a flipped classroom may be intuitively thought to mean, e.g., a crazed classroom, a classroom made full of life again, or a classroom in which the students would be impelled to teach the teacher, it, in fact, relates to the flipping of the times and places at which the introduction with the content and the process of understanding occur. Simply, whereas in the traditional model, the students are acquainted with the content during the class and are requested to deepen the understanding thereof after it, in the flip model, they are introduced to the content beforehand and the comprehension of the content is set to occur during the class through a variety of individual or group learning activities. Learning objects that students are exposed to prior to the class are versatile and may include instructional videos, textbook chapters, professional papers, clinical case reports, or other material. Shifting the in-class focus from the content delivery to the content comprehension, the idea of the flip has had its roots in constructivism, a cognitive theory centered around the idea that each subject constructs a perceptual reality on its own (von Foerster 1972; Glanville 1987; von Glasersfeld 1995). The objectivism of the idea that a single type of delivery would work for all has thus become shunned, and the subjective idealism of the idea that educational focus must be on the fosterage of autonomous construction of knowledge has become embraced.

Studies on the effect of the flipped classroom on student learning widely vary in outcome, highlighting the importance of the instructor, the topic, and the student population. Certain studies have thus shown that students from the flipped classroom score higher on both general course and critical thinking exams compared to the students attending the traditional lecture sessions (Talley and Scherer 2013; Missildine et al. 2013; Mortensen and Nicholson 2015; Hsu et al. 2016). Other studies demonstrate that there is either no statistical difference between the two (Jensen et al. 2015; Eichler and Peeples 2016; Davies et al. 2016; Hotle and Garrow 2016) or that the students from the flipped classroom perform less well than those immersed in the traditional or interactive lecture settings (Bossaer et al. 2016). Studies comparing the flip against the “productive failure” model, where the students ineffectually discuss and try to solve new concepts in the class and then work on understanding them after the class, on their own, showed no significant difference in their gain of procedural knowledge and an even lower gain of conceptual knowledge in the flipped classroom compared to its antipode (Song and Kapur 2017). Interestingly, in spite of the greater exposure to communication requiring critical thinking, students from the flipped classroom are shown to have lower literature searching skills than those from the traditional classroom (Goates et al. 2017), necessitating the incorporation of autonomous literature searches in the flipped classroom to a greater degree.

### The flipped flip

**Fig. 2:**
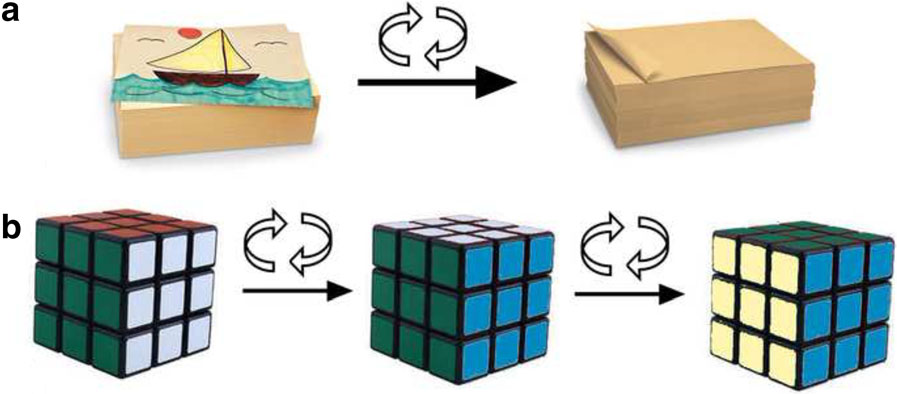
“Flipping the flipped.” Flipping a two-dimensional system, in this case a piece of paper with a sailboat drawn on it, diametrically changes it (**a**), but flipping a three-dimensional system, in this case a Rubik’s cube, need not change what is exposed by it (green surface is still exposed at the front after the first flip) and requires additional flips performed in specific directions to create this change (**b**)

The flip model has been accepted as a diametrical opposite to the traditional lecturing style. Though colloquially perceived as the upside down version of the latter, at this place, I introduce the reader to the idea that the flip shares a number of critical premises with the traditional model. An illustration accompanying this proposition is displayed in Fig. 2 and serves as an instructive analogy. Namely, flipping a two-dimensional system changes what is exposed on its surface by 180^o^, displaying its diametrically opposite side (Fig. 2a). However, life is not two-dimensional; it is, rather, multidimensional, having, say, three dimensions like in the Cartesian coordinate system, four dimensions if we were to describe reality as an Einsteinian, spatiotemporal continuum, eleven dimensions if the string theory was used as to describe it, or *n* dimensions according to the many mathematical models of it. And in such dimensional spaces where *n* > 2, a flip may not “flip” the system and display its diametrically opposite side. As shown in Fig. 2b, the surface of a three-dimensional object facing the viewer, in this case of a cube, may be green both before and after the flip. In that case, multiple flips, that is, variations on a theme transposed on the original theme, may be needed to change what is exposed on the surface of the system and visible to the viewer.

This visual analogy contains a dual instruction. For one, it teaches us that a flip need not fundamentally change the state of the system. The flipped teaching model, as a corollary, may not necessarily be significantly different from the classical model. If this is true, what are exactly the commonalities between the traditional teaching and the flipped teaching? There are many, but the most critical of them is that the teacher in the flipped classroom still authoritatively imposes preset values onto students by independently selecting the material that is to be covered. The flip model, as such, still fosters the reproduction of ideas and information and implicitly teaches conformism, which is incompatible with creative thought.

Second, unlike in the two-dimensional system where a flip of the flipped system brings the system back to the initial state, multiple flips are needed before a three-dimensional system restores this original state. For example, if a cube is flipped thrice along the same rotation axis, a new side will face the viewer after each of the three successive flips and only after the fourth flip will the cube be brought back to its initial state. This is to instruct us that we should not resist to flip the flipped classroom model, fearing that we would bring it back to the traditional lecturing model. We will not, especially in an *n*-dimensional system (*n*~∞) that human knowledge is, where each spin to it brings about an inimitably unique state.

Flip model, therefore, should continue to evolve. If for nothing else, then for the fact that if every instructor were to adopt the same, flip model, diversity would disappear and monotony would settle in. Such may be the fate of many higher education schools and departments today where the flip is imposed on the teachers as the mandatory instructional method. If everybody uses the same teaching method, then it becomes a dogma and critical thinking skills cannot be taught in such an environment. To that end, what follows is the description of a method that evolved out of a personal research in instructional science, which, like most basic research, stemmed more from pure wonder than from a desire to fill a particular application niche. This method is presented here not with an aspiration to impose it onto the global academic community as a universal method. It is presented as a single out of a potentially infinite number of possible “flips of the flip,” that is, variations to the general principles of the flip method of instruction, thus implicitly calling for each reader to continue to evolve one’s own version of the flip, notwithstanding that every flip of the *n*-dimensional object geometrically representing the flip model will by default give a unique instructional state.

Specifically, here I propose a variant of the flip model based on the active involvement of students in searching, finding, selecting, and assembling knowledge from various literature sources into the learning material for the actual class. Because students actively co-create the content together with other students and the instructor, the model is christened as co-creational. Co-creation is best described as a philosophy of thought arguing in favor of a co-creative influence of two sides in determining the appearance of physical phenomena at any scale or domain. Scientific measurements, most notably at the finest, subatomic scales, demonstrate that the properties of the observer and of the observed are intermingled within each physical quality that results from these observational acts. Microscopic analyses, for example, provide images that form as an intersection of the properties and settings of the measurement device and the properties of the measured system (Uskoković 2010). Similarly, our perception is neither objective, i.e., analogous to the way a camera captures the image of reality, nor is it solipsistic, but rather occupies an intersection between these two ontological standpoints. Experience forms at the intersection of the properties of reality, albeit inaccessible to the subject’s senses as-it-is, and the cognitive, biological, and psychological properties and predispositions of the observer (Uskoković 2009). Per the central tenet of co-creation, whatever the creative task we engage ourselves into, we are never alone; there are always at least two sides together involved in creating the created. The main question that this research study aimed to answer was whether the concept of co-creation could be effectively translated from the epistemological to the educational domain.

## Methods

### Student evaluation method

The experimental learning model based on collaborative data mining and presentation was tested in PHS632, a three-credit-hour graduate course on medical devices at Chapman University. The class populace included 8 MSPS/PhD students and was diverse in its ethnic and racial makeup, comprising a Serbian-Slovenian-American instructor and students from India, Iran, Jordan, Nepal, Nigeria, Saudi Arabia, and USA. To avoid the methodological weakness entailed by the comparison of different methods across different student cohorts (Mehvar 2017), a crossover method was implemented and students from a single class were exposed to three different teaching methods sporadically and compared for the outcome. The effect of the model on student learning was compared quasi-experimentally, against two controls, one being the traditional lecturing whereby discussion was supported but no active learning activities took place, and the other one being the default flip model whereby the content was uploaded before the class and the individual and teamwork activities and discussions took place during the class. To assess the student perception on learning, a survey containing 15 questions was distributed to the students two-thirds through the trimester. Each question consisted of a pair of mutually exclusive statements that the students could pick from. To assess the student learning performance, four tests were distributed throughout the trimester, the first two evaluating the didactic and the standard flip models, and the last two evaluating all three pedagogic methods interchangeably. Still, the major emphasis on the latter two tests, which included the most comprehensive, fourth and final exam, was on the co-creational flip. Students were gradually introduced to the latter model, starting from the most familiar and the least active to the least familiar and the most active instructional method. The content assigned for the three midterm tests was not cumulative, but it was cumulative for the fourth and the final exam. In total, this content was similar in the amount for all three methods over the course of the trimester. Fifteen questions in total were assigned to each individual teaching model. Sixty percent of questions required a written input, with the rest being multiple choice (20%), fill-in-the-blanks (13%), and term shuffle (7%) questions. Items were adjusted for the same level of difficulty and ranked 2.0 on average on the scale of 4 cognitive levels per Bloom’s taxonomy (1 - Foundational, 2 - Intermediate, 3 - Advanced, 4 - Mastery). In terms of the total grades, 50% of the students fell in the 70–80% range, and the average equaled 71.8 ± 11.4 (mean ± SD). This comparatively narrow distribution of student performance had a positive effect on the statistical reliability of the study, in spite of the low number of student respondents (*n* = 8). Statistical significance was determined using the student’s *t* test, whereby a difference between the means with the confidence interval, *P* < 0.05 was considered significant. Student intervention policy was at the discretion of the instructor and involved occasional antiplagiarism warnings.

### The co-creational teaching model

**Fig. 3:**
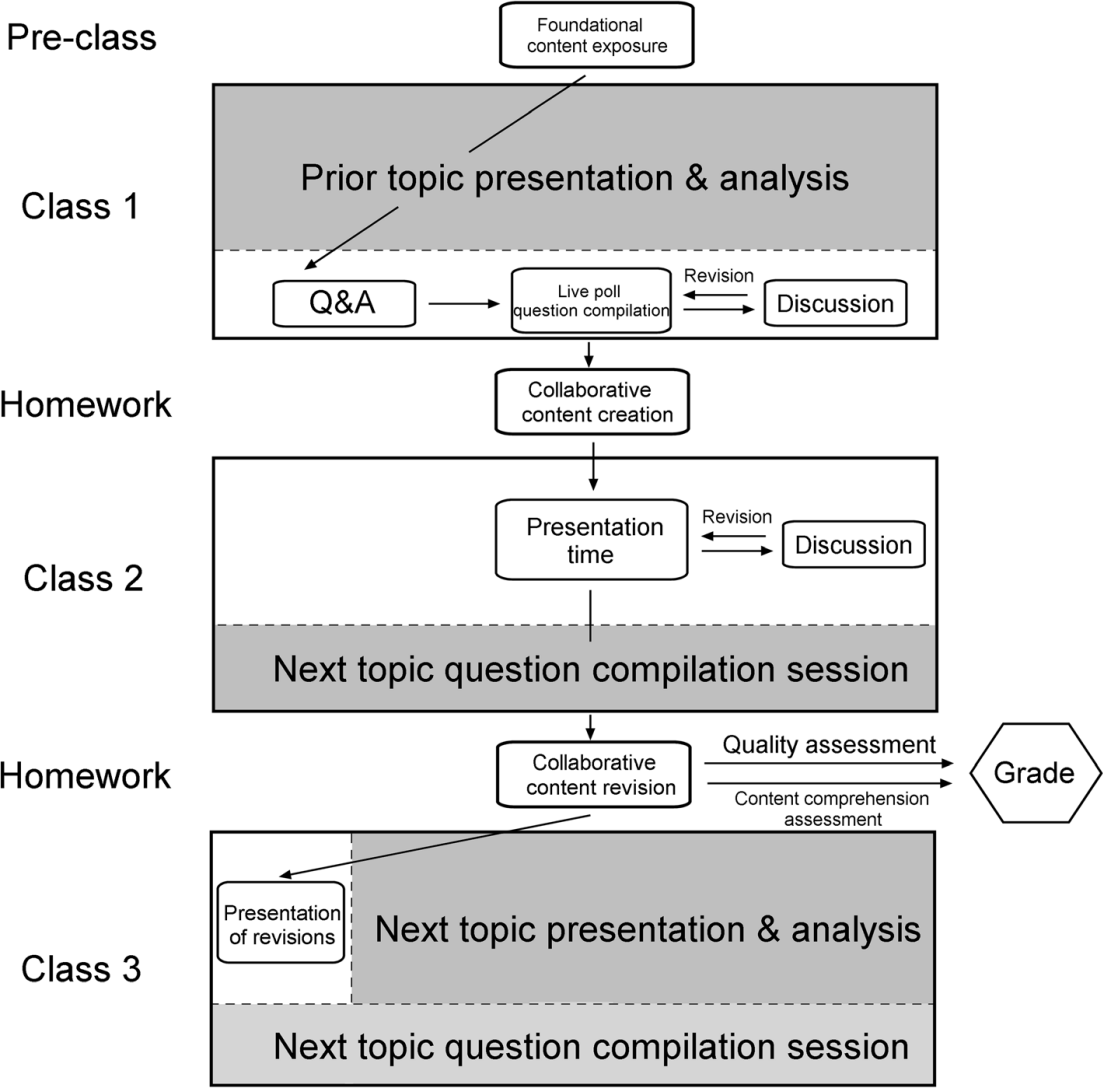
The co-creational model of instruction. The schematic presentation of the co-creational variant of the flipped classroom model

Figure 3 schematically represents the basic instructional steps in the co-creational teaching model investigated in this study. In concert with the flip strategy, selected foundational content in form of slides, book chapters, journal articles, or study guides is made available to students 5–7 days before the actual class. Even though video content has been traditionally associated with the flip model, videos have been avoided in the class implementing the co-creational method because scientific knowledge available in such a format comprises but a tip of the iceberg of the total. Although video content is ideal for use in certain scenarios, readings present students with more in-depth information and also provide a means for developing their abstraction skills. The pre-class content could be uploaded in a form that does not allow its modification, such as Blackboard Learn, but software such as Perusall, an online-based tool for the collective discussion of papers or textbook chapters, could be used as well, depending on the preference of the instructor and the class. When the class convenes and the time for the discussion of this content comes up, a usually brief Q&A session is held to clarify any uncertainties that the students might have, which is followed by a live poll whose purpose is to compile the questions of interest, pertaining to the topic of the lecture, in real time. Students have been shown to provide more insightful and bolder questions when they do so anonymously, in a textual form rather than when they have to orally announce them before the class. For this reason, the question compilation was done through an online social media platform, such as Poll Everywhere or Turning Point. Before the questions are assigned to students, an active discussion is encouraged, during or prior to which the instructor cannot only add his own questions to the board but also rephrase the students’, given the mutual consent, before pasting them onto a collaborative Google Slides presentation. In case more textual presentations are favored by the instructor, a cloud system enabling simultaneous work on Microsoft Word or HTML documents, such as ACS’s Authorea, Wiki pages, Hypothes.is, or video-message incorporating Critique IT, can be used too.

The co-creational model breaks down a single broad topic to multiple questions. The role of the instructor at the question compilation stage is to ensure that the questions fall within the topic; that they, taken together, represent the major points that cover a broad semantic surface, while being individually specific. If questions are too encyclopedic in breadth, the students will be tempted to find answers in common and lowly reliable online sources or may take the dubiously ethical advantage of the fact that the Internet brims with freely downloadable presentations on broad topics. In contrast, when questions require attention to detail, the sources of knowledge become naturally more intricate. In the basic science class implementing this strategy, the students were allowed to use any sources, but earned most points if they were recent journal articles or books, a lesser number or points if they were mainstream textbooks, and the least number of points if they were random internet pages, such as blogs, news articles, company presentations, forums, e.g., Reddit or Research Gate, or encyclopedias, e.g., Wikipedia or Merriam-Webster. Acceptable sources may vary depending on the discipline, the subject, and the depth of study, in which sense more contextual analyses may benefit from looser and less detailed contents.

After the questions are compiled and assigned to students, the work on the creation of the content may begin. The outcome of this work may take different forms and is limited only by the imagination of the class. One possible form is a slide per student, whereby each slide would contain 1–2 images, schemes or tables, and bullet points that summarize the central ideas that the student wishes to retain and share with other students. Depending on the preference of the instructor and the class, the content could be either created during the class individually by students or through teamwork, or it could be created between the classes, as schematized in Fig. 3. The co-creational model, as such, may have varying amounts of homework. As originally conceived, however, it is meant to be supportive of the balance between (a) individual introspection and expression and (b) collective engagement through the spirit of communality because, per inferences derivable from the extension of the concept of co-creational into the social domain, the secret of creativity lies in the simultaneous descent deep into the spheres of one’s consciousness and expansion of oneself into the world (Uskoković 2015), with which one should increasingly identify oneself, provided that the educational approach has lived up to its supreme ethical objectives.

Per Fig. 3, the students are given time between the two successive classes to work on the content, which they add to the collaborative Google Slides presentation and present at the first following class. Discussion ensues, during which missing points are addressed by the class and revisions suggested. Revisions are an integral part of the content co-creation and can take place either during class 2, right after they are being proposed, or as a homework between classes 2 and 3, or both. As shown in Table 2, the quality of the content turned out to have improved when the students had more time to do the corrections and when they did them outside the classroom. To prevent the awkward silence due to hesitation to voluntarily offer a critique of the classmates’ contents, a rotation was reinforced, so that everybody was put on spot to highlight pros and cons and advise addendums after a classmate’s presentation. For a single slide, presentations should be short and adjusted to the class size, typically ranging between 2 and 3 min for a class comprising a dozen students. Individual classes were 2.5 h long, as they allowed more time for presentation, discussion, and in-class content revisions than the 50-min single credit university standards. The final acquaintance with the content occurs during class 3, when students briefly present their revisions to the class (Fig. 3). A farewell to this portion of the total content is waved at this time, albeit temporarily, as the students will encounter it at the first following exam.

Speaking of the exam, what the advantage of crafting a complex content compared to a simpler one is when the students will be tested on its reproduction, students may wonder. To prevent the deliberate creation of a simpler content, which will be easier to comprehend and reproduce, students must be assessed dually: on a content comprehension test and for the content creation quality. The combined performance in these two aspects results in a total grade for that particular segment of the curriculum (Fig. 3). Creation of an overly simple and naïve content may thus earn the student a higher grade on the content comprehension test, but a lower one on the content creation evaluation. In such a way, students are fostered to engage in the co-creation of a meaningful, relevant content, as complex and accurate as possible. A student uneager to implement revisions to a slide after being instructed by the instructor is thus faced with a natural mechanism for the fair assessment of her success in the class. Finally, participation grade, albeit subjective and favoring in theory the often infertile extroversion or simple talkativeness, can be introduced to complement the presentation grade as a measure of the student engagement in the active provision of comments, suggestions, clarifications, and amendments to the collaboratively created content.

### The topical example

The co-creational model is an open-ended model, meaning that it evolves in a new direction each time the subject is being taught. It requires the instructor to give up the solid ground of “complete creative control” over the content of the class and the direction in which it evolves and find comfort on shakier grounds where a sense of uncertainty hovers over it all. In a way, it shares common features with the jazz philosophy, which resists repetition of phrases each new time a tune is being played and relies on relentless improvisation, seeking inspiration from the magic of the moment. Whereas the theme, which may be analogous to the preloaded content, may be the same for each performance, repeating itself at the beginning and the end of the tune, what comes in-between is a one-of-a-kind improvisation that never repeats itself and that may be either collective, as in free jazz, or sequential, involving one member of the orchestra after another, as in classical jazz. A positive feature of this approach is that it facilitates the teacher’s learning, an effect hindered in a system where one falls into the trap of repeating the same key points and walks the same paths over and over again. And a happy teacher, always arriving at new insights in the course of teaching a subject year after year, is an enthusiastic teacher, a teacher able to infect the students with the long-lasting motivation for the subject. On the student side, this improvisatory style allows for the in situ adjustment of the teaching content and style to the student(s) in the class. Given the unique cognitive predispositions and background of each individual, this flexibility of a teaching method and style are prerequisites for pedagogic success. This brings us over to a central point where the co-creational model improves the standard flip, which is by conforming to the age of personalized information, medicine, and other human experiences. This personalized choice of information has been largely ignored by the standard flip model through its provision of prefabbed contents, unadjusted to individual student interests and predispositions. This will be illustrated further with a typical open-ended collaborative content creation and presentation example, the subject of which was the interaction between medical devices and living organisms.

During the question compilation stage, students may demonstrate a wide variety of interests and the co-creational model allows for the adjustment of the content to their curiosity. Today’s graduate courses that aspire to deliver up-to-date theoretical and practical knowledge are intensely multi- and cross-disciplinary in nature. Bioengineering, for example, stands at an intersection of a variety of classical disciplines, ranging from mechanical and electrical engineering to physical chemistry to molecular biology, and it is difficult to predict which student backgrounds and interests will prevail in a class depending on the year or the semester in which a bioengineering course is being taught (summers, for example, may attract more medical students that engineers and graduation tracks usually display spring/fall major degree asymmetry), let alone the institution. Correspondingly, students with a background in basic natural sciences might be inclined to ask questions about the interaction between medical devices and cells at the finest, molecular, and atomic scales. Students with background in mechanical or chemical engineering might wish to learn about the inner workings of particular devices and their functional effects on the organisms. Students from life sciences may be more eager to focus on what happens to cell pathways upon the contact with a device, as opposed to materials science students, who might want to know more about the way in which, say, crystal lattice configurations or surface energy profiles are involved in eliciting specific biological responses. Medical students tend to be curious about the systemic effects caused by the interaction of the body with a medical device, falling back on the medical terminology and notions such as inflammation, infection, and wound healing. If there were social science students attending the class, they might want to dig deeper into socioeconomic factors that define why certain medical devices are offered to patients and the others are not, even though their quality, function, and sometimes even the production costs may be just about the same. Philosophers might ask questions about ethical issues arising from this interaction, poetically inclined attendees may want to hear an inspirational analogy, and so on. Whatever the time allocated to a class focused on this particular topic related to medical devices, there will not be enough time to cover all of this. The adjustment of the content and the discussion to the student interests, offered by the co-creational model, presents a viable solution.

**Fig. 4:**
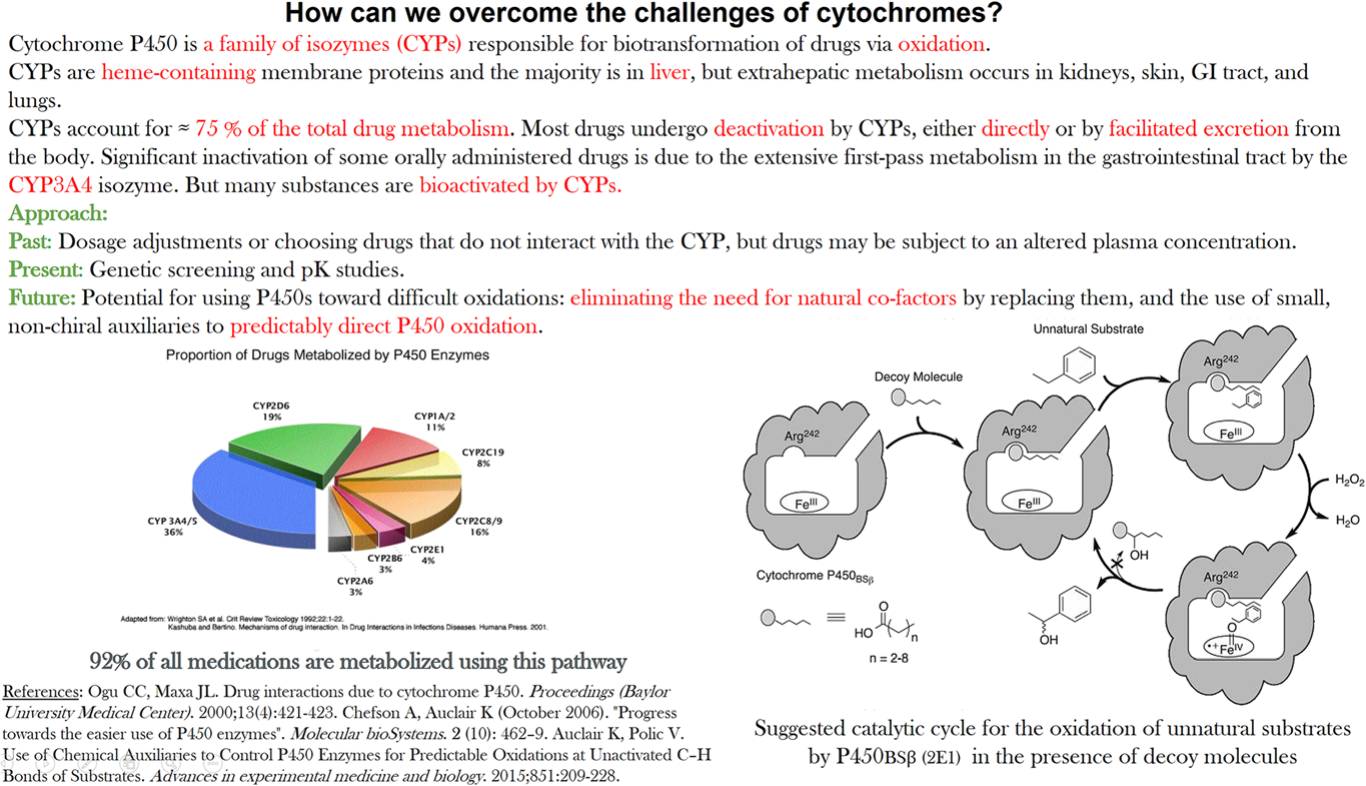
The example of a co-created content. A co-created slide on the topic of overcoming the challenges of the cytochrome

**Fig. 5:**
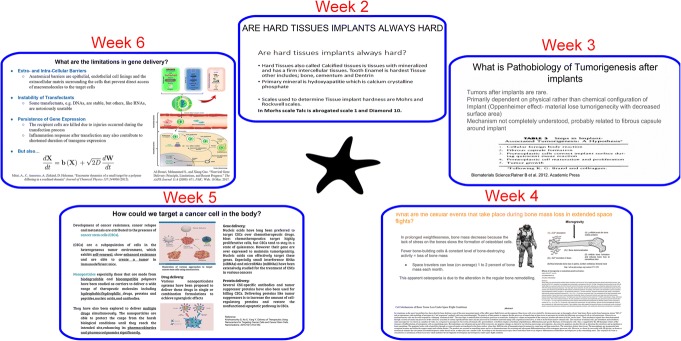
Evolution of the quality of the co-created content and style throughout the course

Table 1 illustrates this variety of student interests by listing questions co-created by the students and the instructor in the medical device class and arranging them into five major categories: general, materials chemistry, biophysics, cell biology, and medical science. Figure 4 displays an exemplary slide co-created by a student and the rest of the class, including the instructor, while Fig. 5 shows the gradual progress in the quality of content co-creation that the students made throughout the trimester. As requested from students, the slide contains relevant background and research information, a scheme and a figure, and correct referencing. As remarked earlier, the role of the instructor is to add, modify, and select questions, given the consent of the class, and make sure that they, taken together, cover an optimally broad semantic surface. Connections between semantic points corresponding to individual questions can be always drawn with a little bit of imagination and an advised activity consists of assigning an individual student rotation or teamwork to create a concept map whereon relationships between individual questions or answers thereto are projected.

**Table 1:** Questions compiled during an in-class polling session on the topic of interaction between medical devices and biological systems and classified depending on the discipline

Type	Question
*General*	How do we classify different types of interaction that might occur? What is the duration of the device integration process?
*Materials chemistry*	Are natural biomaterials more interactive than the synthetic ones? Is it BIOmaterials or bioMATERIALS when it comes to defining this interaction? Does the conformation of adhesion proteins change depending on the crystal face dominantly exposed on the device surface? What surface properties decide if the device will be integrated or rejected? Can topography be designed to repel bacteria and promote adhesion of host cells?
*Biophysics*	Why do hydrophobic surfaces bind more protein than the hydrophilic? Can entropic factors dominate the protein-material interaction energy term? Why is the initial adsorption rate often smaller than expected from diffusion coefficient values?
*Cell biology*	What are the different cell responses depending on whether the cell has the affinity for the surface or not? How do we investigate the effects of substrate surface chemistry on monocyte adhesion, macrophage fusion, and foreign body giant cell development? What are the microenvironmental and the systemic responses to medical devices? What types of cells are most amenable to surface interaction? How can the interaction between cells and the surface of the device affect wound healing? How is hemocompatibility created in a device and how does it differ from other cell type compatibilities?
*Medical science*	When does the interaction between the cell and the medical device result in an inflammatory response? What are the standards, specifically positive controls, used in studying the integration of the device? What are the age and physiological barriers in the interaction of cells and tissues with medical devices? Could a silent, asymptomatic interaction lead to severe problems? Does pyrogenic reaction, i.e., fever, invariably follow the implantation of the device? How can we prevent the infection and granulation tissue complications after the insertion of a medical device? What is the pathobiology of tumorigenesis triggered by medical devices? Which of these toxicity tests provides most valuable information––systemic, acute, subacute, or subchronic?

## Results: student response to the teaching method

Both the student performance on tests and their perception of learning using three different teaching models implemented in the class were assessed quasi-experimentally. Examples of test questions matched with cognitive level categories and deliverable using the ExamSoft platform are shown in Additional file 1: Figure S1. The three teaching models compared were:

1. Traditional lecturing (discussion supported but no active learning exercises);
2. Standard flip (content uploaded before the class and individual and team-based learning activities organized during the class);
3. Open-ended collaborative co-creation of presentations on questions of interest to the whole class.

**Fig. 6:**
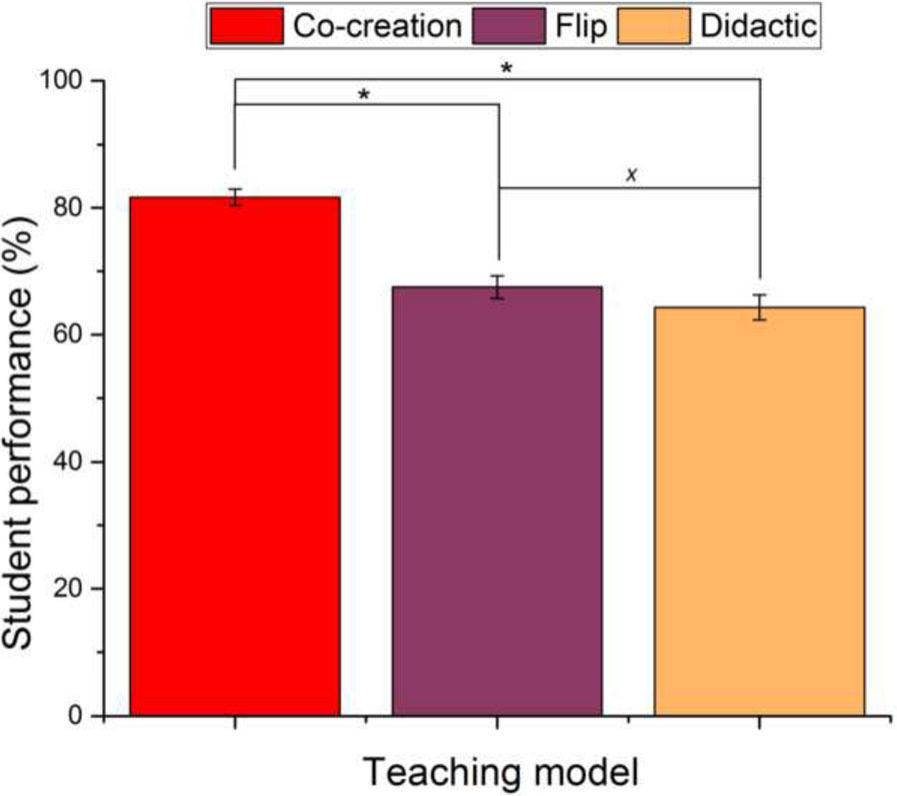
Instructional outcome. Student performance on exams (*n* = 8 participants × 15 items for each of the three methods) testing for content covered using the different teaching models implemented in the class. Data points are shown as averages with error bars representing the standard error of the mean. Statistically significant difference (*P* < 0.05) between different sample groups is marked with an asterisk. Statistically insignificant difference (*P* > 0.05) between different sample groups is marked with *x*

Results demonstrating a better student performance when tested for knowledge covered in the co-creational section of the class as opposed to either the traditional lecturing portion or the standard flip one are shown in Fig. 6. The great majority of students, 87.5% of them, scored better on the test for knowledge covered in the co-creational segment of the course than on either the didactic one or the standard flip one, with the average difference between them being 17.34% (*P* = 0.022, *r* = 0.948) and 14.17% (*P* = 0.04, *r* = 0.933), respectively. The students performed better on the standard flip portion of the test than on the didactic one, but the difference was negligible (3.18%) and statistically insignificant (*P* = 0.68, *r* = − 0.457). It is interesting that the questions requiring a narrative as an answer were better answered in the co-creational section of the test than in the didactic or the flip one, indicating a greater fluidity and confidence with which students can express ideas after being exposed to the co-creational experience.

When it comes to the student perception of learning in the class, the results on which are shown in Table 2, the students preferred both the co-creational model (Table 2a) and the classical flip (Table 2c) over traditional lecturing. However, the students had come to the class with an already established preference for (inter)active learning over the didactical classroom experience (Table 2l), which may explain their preference for the two active models. They were divided in terms of which they liked more: the standard flip or the co-creational flip (Table 2b). However, they felt that they learned more using the co-creational model than using either didactic lecturing or the standard flip (Table 2d–e). As one of the key premises of the co-creational model is the instructor’s active engagement in content creation and, more importantly, learning, it should be noted that I heard a greater number of new ideas and that the opportunity for the enrichment of my knowledge was larger during the co-creational sections of the class than during the traditional lecturing or the standard flip model.

**Table 2:** Statements from the survey on the perception of learning using different teaching models with the level of agreement ≥ 50%

Statement	Level of agreement (%)
a. I liked the open-ended collaborative content creation and presentation model^*^ in this class more than the traditional lecturing	87.5
b. I liked the open-ended collaborative content creation and presentation model in this class more than the standard flip model	50
c. I liked the standard flip model in this class more than the traditional lecturing	75
d. I feel like I learned more with the open-ended collaborative content creation and presentation model than with the standard flip model	62.5
e. I feel like I learned more with the open-ended collaborative content creation and presentation model than with the traditional lecturing	62.5
f. I preferred working on the content creation as a team than working alone	75
g. I would prefer working on the content creation in the class than as a homework assignment	55.5
h. Content and learning quality were higher for homework assignments than for in-class preparation	62.5
i. Open-ended collaborative content creation and presentation model motivated me to pursue this subject after the course more than traditional lecturing	75
j. Open-ended collaborative content creation and presentation model motivated me to pursue this subject after the course more than the standard flip	50
k. Open-ended collaborative content creation and presentation model was more open to intrusion of moral instructions than traditional lecturing	75
l. In general, I prefer (inter)active learning over traditional lecturing	75
m. I feel like the teamwork did not have more of a community-building aspect than the individual work on presentations	50
n. Open-ended collaborative content creation and presentation model is more suitable for impressing future job employers than traditional lecturing	71.4
o. Open-ended collaborative content creation and presentation model is more suitable for impressing future job employers than the standard flip	62.5

*The open-ended collaborative content creation and presentation model is synonymous with the co-creational model

The students felt that the co-creational model motivated them more than lecturing to pursue the subject after the course (Table 2i), but were again divided when it came to comparing the effects of the standard flip and the co-creation on this motivation (Table 2j). They felt that the immersion in the co-creational classroom would help them gain knowledge and achieve skills most useful for impressing the future job employers in high-tech industry or academia (Table 2n–o). This may speak in favor of their gaining more confidence with respect to presentation of knowledge than using other models tested. Equally, a sense of intellectual empowerment consequential to learning how to search for right content across a vast literature landscape may have been an outcome of immersion in the co-creational classroom. The students were divided in terms of whether they liked in-class or in-between-class work on content creation (Table 2g), but felt that they learned more if they did the major, initial part of content creation as a homework (Table 2h). They also felt––and the instructor could attest to this––that the quality of the content was drastically better when the first round of its creation was performed individually by the students (Table 2h), in-between the classes, as schematized in Fig. 3. In spite of the fact that the students preferred working as teams over working alone (Table 2f), they did not feel as if the solitary first round of content creation had a detrimental effect on community building (Table 2m), presumably because of a great deal of discussion and content revisions that take place through everyone’s involvement in the classroom. An ideal number of students in teams for the in-class content co-creation was 2.7 ± 0.8 (mean ± SD). Finally, the students felt that the co-creational model was more open to the intrusion of moral instructions than traditional lecturing (Table 2k).

## Discussion

### Formative nature of the co-creational flip

The goal of this study was to provide the basis for an innovative variation to an already existing instructional model, namely flip. The study is built on an aspiration to provide a seed of novelty and a rudimentary proof of concept, but then leave space for the involvement of a broader effort to shape the model into a more defined form. Simultaneously, the model should not relinquish its open-ended character and should allow for the ad hoc adjustment to the individual predispositions of the instructor and the class at hand. This inherently conceptual nature of the study is consistent with the broader philosophy employed by this instructor in the lab: the provision and the proof of original concepts, often falling in the domain of the glass bead game, is an essential aspect of research conducted in it. The lab counters the trend of increasing industrialization of academic research and strives to support a space for the exhibition of science in its purest form, which shares more in common with arts than with contemporary R&D entrepreneurialism. What differs this vintage vision of science from its industrial, applicative counterpart is the breadth entailed by the quest for proofs of concepts vs. the narrowness of focus brought about by excessive optimization and the overemphasis on precision. This is what brings pure science close to arts too, if not merging them into an inseparable unity. In the spirit of the conceptual arts, this study, like science done in the aforementioned laboratory, implicitly questions the climate and the predominant practices within the modern science. One of these critiques implicit in this study pertains to the excessively technical style with which educational studies are being conceived and presented, being in disparity with the educational goal to inspire and be esthetically lavish. The design of this study, thus, concords with the aspirations intrinsic to the instructional model presented on, which is to be both informational and inspirational, to which end this report assumes a more poetic style of expression than typical for technical papers, especially toward the beginning and the end, thus reflecting the ideal path for the evolution of education from the history to the present to the future. The main challenge for the design for future research and refinement of the method proposed here is tied to the free, undefined nature of the model at its multiple levels. However, this improvisatory, open-ended character, endowing the model with formative limitlessness, increases the number of options and degrees of freedom for its evolution and, thus, per von Foerster’s constructivist standard (von Foerster 2010a), satisfies the demand for ethicality.

### Co-creational flip and the four pillars of F-L-I-P™

The co-creational version of the flip model conceived in this formative project overwhelmingly conforms to the four central principles, so-called pillars of the flipped learning (Flipped Learning Network 2014; Anon 2014): flexible environment, learning culture, intentional content, and professional educator. Complying with the F.1 principle, broad spatiotemporal frames are maintained to allow their inhabitance by the students and their free conversations for the sake of anarchic arrivals at unpredictable collective insights. Reflections on learning and excursions into the metalogical realm are also naturally encouraged in the co-creational classroom. As for F.2, ad hoc adjustments to content and conducted activities are constantly implemented, albeit not by the instructor per se, but by every attendee of the class. Finally, in concert with the F.3 tenet, instructional incentives are crafted to the needs of each student individually and are, as such, unique and unrepeatable, helping the instructor to avoid falling into the trap of prefabbed content delivery. The flexibility of content creation is introduced as an additional facet of flexibility in the co-creational model. As for L.1 norm, the centrality of the teacher’s presence is being shunned and everyone’s opinion is highlighted as equally relevant, while the clashing of ideas, not egos, is encouraged, all for the purpose of the dialectical deduction of new insights to both the class and the instructor. Naturally, the intentional content pillar is the central point of diversion from the flip as defined by the Flipped Learning Network. Contrasting the idea that the instructor alone is responsible for the creation or curation of relevant content for the students, students are actively engaged in every step of the content creation process. In that sense, the co-creational model converts the “intentional” to “interactional” in this third pillar of F-L-I-P™. As for the fourth pillar, the professional educator one, the model complies with all three of its subprinciples by having the instructor available in real time to provide the feedback to the students (P.1), attentive to the atmosphere in the class to inform future instruction (P.2) and, more than anything, collaborative in and out of class for the sake of continually evolving and perfecting one’s practice (P.3). At the same time, however, through its anarchistic premises, the co-creational model questions the notion of professionalism per se, disliking its stiff and affected undertone and favoring its opposite, a.k.a. amateurism, all along with its connotation of intuitiveness, of inclination to incessantly improvise and of courage to live up to the Latin roots of this term, *amare*, meaning “love,” the first and the final point of good educational practice.

### Co-creation as an open-ended dialogical education

That conversation in literal or abstract terms is required to enable learning has been known since Aristotle’s proposition of the syllogism rule (Aristotle 2009): namely, two disparate statements need be posed side by side and allowed to semantically cross-fertilize before a new idea or an insight can be reached. This principle was translated to a more palpable pedagogic realm by the cybernetic conversation theory (Pask 1975), and today presents the mainstream approach to learning adopted by academic institutions. Co-creational addendum to this basic dialogical model lies in its opening the space for students to actively and semi-autonomously construct knowledge in place of merely discussing it. One practical advantage of the co-creational teaching method is that the self-created content is the stickiest of them all, helping the long-term retention of knowledge. It is for this autodidactic reason that recreating content through written/oral expression improves the likeliness of its memorization compared to its simple abstract recollection. Another essential advantage of this method is that it teaches how to autonomously question, find, assess, and express knowledge, which paves the way for originality and creative thought. If research is considered the pinnacle of scholarly work, then the co-creational model can be envisaged as a bridge between the classroom and the research. And since prolific research thrives in free-minded settings that encourage questioning everything, the co-creational method aspires to create the spirit of freedom in the classroom, which acts as a double-edged sword. In extreme cases, it poses a threat of imminent dissipation of the learning momentum of the class, dropping the learned content down to insufficient levels. At the same time, it creates a state of omnipotent vacuity wherefrom monumental creative powers can start to brew. After all, if the ascent to the peak of the Bloom taxonomy pyramid (Bloom 1956; Anderson and Krathwohl 2001), where creativity awaits the ascender, is indeed the ultimate goal of education, then a model like this one, engaging students into autonomous search for knowledge, is analogous to propelling them in that direction.

The optimal interference of the instructor in the content co-creation stage should be neither too extensive nor too miniscule. In case the syllabus predicts 3–4 content comprehension exams interspersed throughout the semester, it may be recommended that presentations for the first exam are minimally adjusted and allowed to look somewhat sloppy, containing ambiguous phrases, as students would realize as they prepare for the exam that there are benefits of correcting each other lest the learning process and the test performance be hampered. It is thus that they spontaneously realize how essential their involvement in suggesting amendments and corrections to the content created by their fellow classmates is. This inherently antiauthoritarian stance can be proposed as a central premise and prerequisite of learning in the co-creational classroom.

This mention of antiauthoritarianism brings the discussion over to the central moral point from which this model has arisen. This point advocates creative chaos, anarchy in a truest sense of the word, as a pathway to the most prolific classroom experience. Directing people toward a specific action or a thought process is inherently authoritarian and toxic as such, the solution to which may be a learning setting where everybody’s opinion resonates with equal relevance. Paralleling the co-creative involvement of every class attendee is the necessity to be out of compliance with the syllabus for most of the time. To be ready to depart from a prefabbed blueprint for our actions, including a preconceived syllabus, is to foster a wide-awake perception among the class participants and open broader gateways for the inflow of creative thought than possible in an authoritarian classroom where off-script activities are strongly prohibited and everything proceeds inertly, in a preplanned manner.

The word co-creation denotes a mutual involvement of the instructor and the students in the content creation process, but it connotes the creative aspect of this process in the relationship between the student and the existing body of knowledge too. It implies that the students are to, ideally, pick up on this existing body of knowledge and add something of a personal creative zest to it. Since genuine creation of new knowledge is impossible for most students, the model allows the content creation process to be adjusted to a greater degree of invention and discussion of original, new ideas for a graduate class and to provision of relevant answers to foundational questions and using the right literature sources for an undergraduate class. Also, not only can a middle ground between these two be adopted, but an alternation between them throughout the semester is possible too. Which brings us over to another major positive feature of the co-creational model, which is its stylistic flexibility and the possibility of utilizing utterly different forms of content co-creation from one class to another. Student learning is multifarious and influenced by a number of factors (Koedinger et al. 2013), whereas variety is a driver of learning and discovery (Dörnyei 2001; Sanchez 2001). Therefore, regardless of the model, if it becomes repeated without any change throughout the course of the entire semester, it may create monotony and a sense of boredom, which might reduce the motivation for engaging in autonomous search for knowledge per se, being the profoundest take-home point for students exposed to this teaching method.

As schematized in Fig. 3, the co-creational model fosters parallel learning, as resulting from the fact that each topic is spread out across three consecutive classes and three separate topics are analyzed in each class. Students are, therefore, required to mentally process multiple sections of the curriculum simultaneously rather than sequentially, as in traditional classroom. This promotes the awareness of interconnectedness between individual topics within the subject of the class and the perception of the latter as a whole rather than a disconnected mishmash of ideas. This method also conforms to the current tendency of students to have their attention dissipated in multiple directions as a result of a greater exposure to information contents from multiple sources than ever before in history. In a way, this presents a way of transforming a colloquial threat for the efficient absorption of knowledge into an opportunity for learning. This spread of content analysis over three classes, even if they be 3 h long each, also prevents the “in one ear, out the other” effect often seen in the sequential topic coverage in traditional curricula. In that sense, the model also counteracts the contemporary demands to provide an immediate feedback to student answers and uses technologies to produce the opposite effect of extending, not shrinking, the content coverage, all for the sake of deepening the knowledge acquisition process rather than shallowing it.

One feature of this model, entailed by its open-ended character, is that a wrap-up of what was learnt, at least at the effable level, is possible toward the end of the class, but learning objectives at its onset are not possible given that the class knows not in what direction the co-created content of the lecture will evolve. Here, we arrive at another critical similarity between the traditional lecturing and the flip model in the form in which it is predominantly taught today. It is that of insistence on clearly delineated learning objectives often not only prior to the class but also prior to the planning of the course in question. In reality, however, the classroom experience, if conceived as a microcosmic reflection of life, should be free of learning objectives. This is especially so in the active learning process during which students and teachers coevolve in an improv manner toward unexpected insights that are invariably unique and differ from one class to another (Sawyer 2000) and, in fact, in any scenario where the development of critical thinking skills is favored over the simple regurgitation of facts and principles and where education is approached as alive and ever-changing. Presetting learning objectives is usually followed by conceiving a path set in stone that would lead to them, signifying the excessive structuring of educational efforts and, in reality, imposing principles, paths, and ideas onto students instead of fostering their own independent arrivals thereat. This inordinate structuring also discourages the liberal, free-spirited thought, the driver of the scientific and cultural progress for millennia, and enforces dogmatic absorption of opinions, alongside promoting obedience to authority and inconspicuously instructing students to be just like the authority they once obeyed: autocratic rather than anarchistic, inclined to tell others what is right and what is wrong and what paths ought to be followed while ignoring the toxic influence of such an epistemic arrogance. Therefore, in the co-creational classroom, no words need be said about the content of the class at its beginning and it is allowed to adopt a free form, with its flow being like a river, evolving in curled lines, unpredictably, by bouncing off the banks of the instructor’s and the students’ ideas and moods of the moment.

### Assessment in the co-creational model

Figure 6 illustrates the positive outcome on tests, namely the ability of students to retain the information covered using the co-creational model better than using the standard flip or traditional lecturing. Students also had a positive perception of the method, as compared to the traditional lecturing and the classical flip (Table 2). These correlations should be taken up with a dose of caution, not only because studies on bigger student cohorts and more diverse topics, involving also multiple instructors, each bringing one’s own interaction style to the classroom, must be used before more confident comparisons between models could be made, but also, more obviously, because educators should not let what students like––rather than what they need––be the determinant of success of an educational method, and, less obviously, because any correlations between the student success on exams testing for content reproduction and the excellence of the teaching method are erroneous. If the latter correlation was assumed to be correct, then the traditional alternation of lecturing and lab exercises would prove to be the best model, at least in natural sciences, and education would never evolve past it. As a matter of fact, the flipped classroom, when evaluated for success in knowledge dissemination, does not always prove to be better than the old model (Zainuddin and Halili 2016). The lower content quantity coverable in the classroom during the course presents a downside of all active learning models compared to the traditional didactic models (Uskoković 2017) and is shared by the variant of the flipped classroom elaborated here.

Scholarship of teaching and learning has a benevolent goal, and its methods are bound to lead to innumerable insights on the science and art of interpersonal communication, but there are definite dangers tied to consideration of something that belongs to the domain of arts and humanities as if it was empirical and treatable as a quantitative science. For, in the end, neither the student perception nor the performance on tests can be reliable indicators of the excellence of a teaching method. Education comprises a number of immeasurable elements, ranging from the inspirational to motivational to ethical and aesthetical, which are all likely to be enriched with the use of dialogical educational models that allow for the sense of community and the moral responsibility entailing it to be built. In other words, not all is in grades, and perhaps whatever the educational system evaluation we conceive of, it will fail to correlate with its excellence. And yet, there is a dangerous trend of increasing complexification, not loosening, of assessment strategies that grips the flipped classroom as I write.

Although experience does show that students do not come overwhelmingly prepared to the class when they are given the task of getting acquainted with the flipped material provided beforehand, it is often neglected that the use of gradable quizzes to open the class with and force the students to study the given material thereby is offensive to both the students and the instructor, the former because it sends the message that students, potentially lazy and immoral, are to be mistrusted and the latter because it implies the instructor’s conciliation with the fact that nothing more inventive could be delivered as an incentive to study but a cold and punitive mechanism distributed in the form of a quiz, usually with the help of yet another cold mechanism: a computer. And since every instruction conveys implicit, hardly effable points in addition to the explicit ones, we could conclude that every educational method that relies on assessments is inherently corrupt. For, whatever the communication in question, the most significant information transmitted by it is not tied to its explicit and capturable content, but emanates from its implicit and ineffable elements (Wittgenstein 1918; Winograd and Flores 1987; von Foerster and Poerksen 2002). In this case, these implicit lessons amount to the possibility of raising generations that will consider only what is graded as important and only what is monetizable as valuable, while ignoring the common wisdom that holds that the most valuable things in life come priceless, immeasurable, as it were. Nowhere less importantly, the students are being taught by means of exposure to an overly structured system of assessment to approach problem-solving by waiting for instructions, like robots, rather than feel internally motivated to probe systems with their own curiosity. Moreover, in active learning frameworks, this obedience achieved through grading is often gamified to increase the student satisfaction and appeal to the videogame generation, resembling the recent attempts of some Orwellian governments to create a point-reward credit system for monitoring and assessing online social network participation and offering/denying social services correspondingly (Botsman 2017).

Regardless of the form it takes, assessment in the classroom disseminates the false premise that the ascent from the base to the apex of the Bloom taxonomy pyramid is somehow ratable, when in reality the higher we ascend, the greater is the impossibility of measuring the success of one’s effort to ascend. When one adds on top of all of this the fact that *learning* in the classroom of the most superb educators presents only one side of the coin, the other side of which is *unlearning*, in the sense of questioning and subjecting to rigorous scrutiny all that has been learned so far in preparation to become a critical thinker and an opposite of a bigoted, robotic performer of SOPs, a dynamic state of acquisition and erasure of knowledge is arrived at, apparently neither quantifiable nor even vaguely gradable. Two choices thus appear before the higher education instructors aspiring to pave way for the climbs to the true peaks of the Bloom taxonomy, but being forced to grade their students: (a) to give up their academic posts and substitute classrooms with open skies under which Socrates taught or (b) to convince students in futility of the use of tests as the grading tool and immerse them in the impressions of such an ethical and esthetic splendor that they would no longer care about the way any judges in life would grade them.

Models, therefore, must be conceived that would foster student learning independently of superficial assessment. The model described here has attempted to do so, albeit within the limits of grading typically requested in a university setting. Proposing a model devoid of any grading schemes is out of question in the current academic climate of, as we see, increasingly excessive and extensive assessments. Though badly sought, it would be surreal and predestined for failure because of its practical disconnect. But designing and implementing models that are conducive to minimization of grades and structured so that they gradually prove their obsoleteness represents a potentially more viable strategy. This idea is an umbrella under which the conception of the co-creational learning model has taken place. And so I end this on a note that is but a prelude to a silent call for the reconsideration of grades and their annihilation on a bright future day, allowing the teachers to break the barrier of mistrust that assessment imposes between them and the students and become what the dream of the purest among them has always been: to be not information conveyers, but inspirers; to be not instructors, but guiding stars; to be not judges, but the savers of the world. It is a note that the co-creational learning model whistles through a quiet night.

## Conclusion: co-creation as an incentive to poeticize education

The teacher is no longer merely the-one-who-teaches, but one who is himself taught in dialogue with the students, who in turn while being taught also teach. They become jointly responsible for a process in which all grow. In this process, arguments based on ‘authority’ are no longer valid; in order to function authority must be on the side of freedom, not against it. Here, no one teaches another, nor is anyone self-taught. People teach each other, mediated by the world.

Friere, 1993.

Benefits of mutual teaching in the classroom, the instructor’s of the students and the students’ of the instructor and other students, are numerous, but they collectively stem from the restoration of a sense of engagement of everyone in discovering and creating new knowledge, the beginning and the end point of scholarly work. There, the implicit goal of this model is to produce a deeper knowledge-centered classroom experience compared to the standard flip, while sailing by some of its dangerous student-centered and teacher-centered attractors, including the mediocritized learning content and the intrinsically uncreative coin having authoritarianism engraved on one side and conformism on the other, respectively. The anarchic dissipation of the sense of authority and its equal distribution across the entire classroom may lower the amount of the curricular content and make it more chaotic, but the effect on the students’ creativity, the ultimate destination of educational paths, can be multiplied, albeit immeasurably. The Waldorf system was conceived with this co-educational goal in mind, which its founder, Rudolf Steiner, believed would change the academic dissemination of knowledge for good (Steiner 1924), not knowing that this noble ideal of his would become squeezed between two fatal eras of autocracy in German history, leaving us with a question of whether the world has learned from the failures of the intellectual despotism of many Dr. Caligaris or is once more bound to fall into the trap of instructional neo-fascism. Still, liberated from the drag of the mainstream thought, countless were educators that challenged the totalitarianism of “the great didactic” (Comenius 1907) throughout the history, from Socrates to Salman Khan. As if being inspired by M. C. Escher’s two hands drawing one another in concert with the “as we create, we are created” adage, portraying the necessity of being changed from the inside that comes together with the process of changing another and, therefore, the need for the teacher to allow oneself to be educated by those that she educates, a Norwegian musicologist came up with the following principle to guide the most prolific educational efforts: “Learn from children – and children will learn from you” (Bjørkvold 1989).

The act of giving parallels the act of seeking and the creation always creates its creator as much as it is being created by the creator. “If you desire to see, learn how to act,” von Foerster’s aesthetical imperative asserts (von Foerster 2010b), whereas to be open-minded, ready to receive, is a prerequisite for the expressions brilliantly tuned to the occasion to emerge out of one. It is as if a law of action and reaction is valid in this context, implying that something has to be given away in order for an insight to be reached and grasped and vice versa. The same, undoubtedly, applies to every path of progress in life: the moment we stop yearning to grow and wishing to reach the most sublime star in the sky of our mind and bring it down to Earth, to share with others, the doors for our own advancement and for inspiring another become suddenly shut. But should we strive to spin the wheel of personal growth that resides in the core of our heart and make it a carousel of wonder and love that will launch us to the farthest and the most magnificent star of thought, the chances for surprising others and ourselves with the inspirational insights reached along the way would skyrocket. This sea of starry surprises is what the co-creational method craves to replicate in the classroom. For, to shed stardust of surprises on the wings of resistance to conform to expectations is a prerequisite for sowing the seed of creativity, of that antiauthoritarian power wholly focused on finding and fixing a flaw in the fabric of human thought, as revolutionarily and anarchically as it can get.

**Fig. 7:**
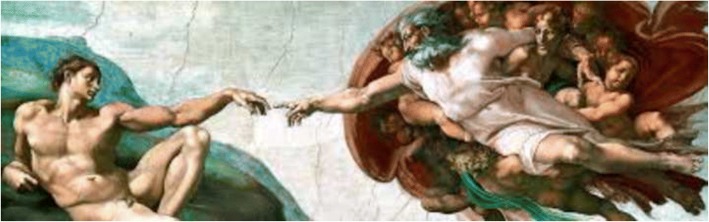
The creation of Adam. The ultimate goal of education so often forgotten in the modern times: transmission of the invisible sparkle that inspires the soul of the student. A wonderful insight it is to realize that antiauthoritarian, inherently anarchic co-education, whereby the teacher and the student rapidly swap roles in pirouettes, inspiring one another in turns, is a means for the seamless accomplishment of this aim. The image is not subject to copyright permission

After all, if creativity, that unteachable, ineffable, and elusive endpoint of educational efforts, inevitably but unperceivably sprouts from the seed of love, of cordial care for another, and if love withers without communication, then only through communication based on the openness to mutual change can we help others land on the top of the pyramid that the Bloom taxonomy, the ultimate destination of the art of teaching is, and only via seeing the world through the eyes of another may we teach and learn in the most effective way conceivable. “Love and do what you will”, St. Augustine of Hippo prophesied (Mersch 1938), and although the Bloom taxonomy is standardly portrayed as a pyramid, the creative top of which is thought to be reachable only stepwise, by climbing from its bottom upwards and passing through the successive stages of remembering, comprehending, applying, analyzing, and evaluating, love can teach students how to fly, and if they fly, they can reach this peak whereat creativity lies by bypassing the regular route. If anyone holds that this is impossible, direct them to children, those epitomes of creative thought, of unending streaks of discovery and infinite wonder and, thus, the beauty of being: they know not how to assess, they have barely any fundamental knowledge, they remember little and judge even less, yet they are utterly creative. Now close the door quietly and listen carefully because this will be my confession: since I, as a teacher, am interested in nothing but the peak of this pyramid, my first and foremost goal in the classroom and beyond is to inspire and open the mysterious mental channels leading thereto before the listeners, passing through which they would become likened unto children and reenter the paradise lost long ago as fast as the interstellar traveler from Ivan Karamazov’s dream arrived at his millions of light years remote destination: in the blink of an eye (Dostoyevsky 1999). Albeit forgotten in these modern times in favor of the focus on the palpable, practical and effable, the ultimate goal of education is to transmit an invisible sparkle of inspiration that enlivens the spirit, that enkindles wonder, and that elicits dedication to a creative work that stems from cordial care for the frailties of this world (Fig. 7). Therefore, to love another is to help one attain these apices of scholarly growth through an act of magic, whereby love, remember, always, is about listening, in the spirit of Mary of Bethany (Luke 10:38–42), as much as it is about erupting quietly with invisible light that blesses and beautifies, like a burning star of the night sky. That––it should never be forgotten––is where the road of co-creational education, undyingly poetic, must lead to.

## Additional file

Additional file 1: **Figure S1.** Examples of test questions matched with the cognitive level categories and deliverable using the ExamSoft platform. **Figure S2.** Timeline of the evolution of the form of the co-created slide on the topic of overcoming the challenges of the cytochrome from after the homework stage, where the student worked alone on the content creation (a), to after its revision in response to the input received during the class (b), to the final adjustments for clarity after the second revision (c). The first round of revisions referred to correct referencing, additional details, and a complementary figure. Other slides co-created in the class underwent a typically greater degree of change during the same timeline progression from (a) to (c). **Figure S2.** (DOCX 1274 kb)
