# Supplementary material for: Flipping the flipped: the co-creational classroom
Source: Res Pract Technol Enhanc Learn. 2018 Jul 31;13(1):11. doi: 10.1186/s41039-018-0077-9 (PMC6294219; doi:10.1186/s41039-018-0077-9)
Supplement: Supplementary file 1 — Figure S1. Examples of test questions matched with the cognitive level categories and deliverable using the ExamSoft platform. Figure S2. Timeline of the evolution of the form of the co-created slide on the topic of overcoming the challenges of the cytochrome from after the homework stage, where the student worked alone on the content creation (a), to after its revision in response to the input received during the class (b), to the final adjustments for clarity after the second revision (c). The first round of revisions referred to correct referencing, additional details, and a complementary figure. Other slides co-created in the class underwent a typically greater degree of change during the same timeline progression from (a) to (c). Figure S2. (DOCX 1274 kb) [file 41039_2018_77_MOESM1_ESM.docx]

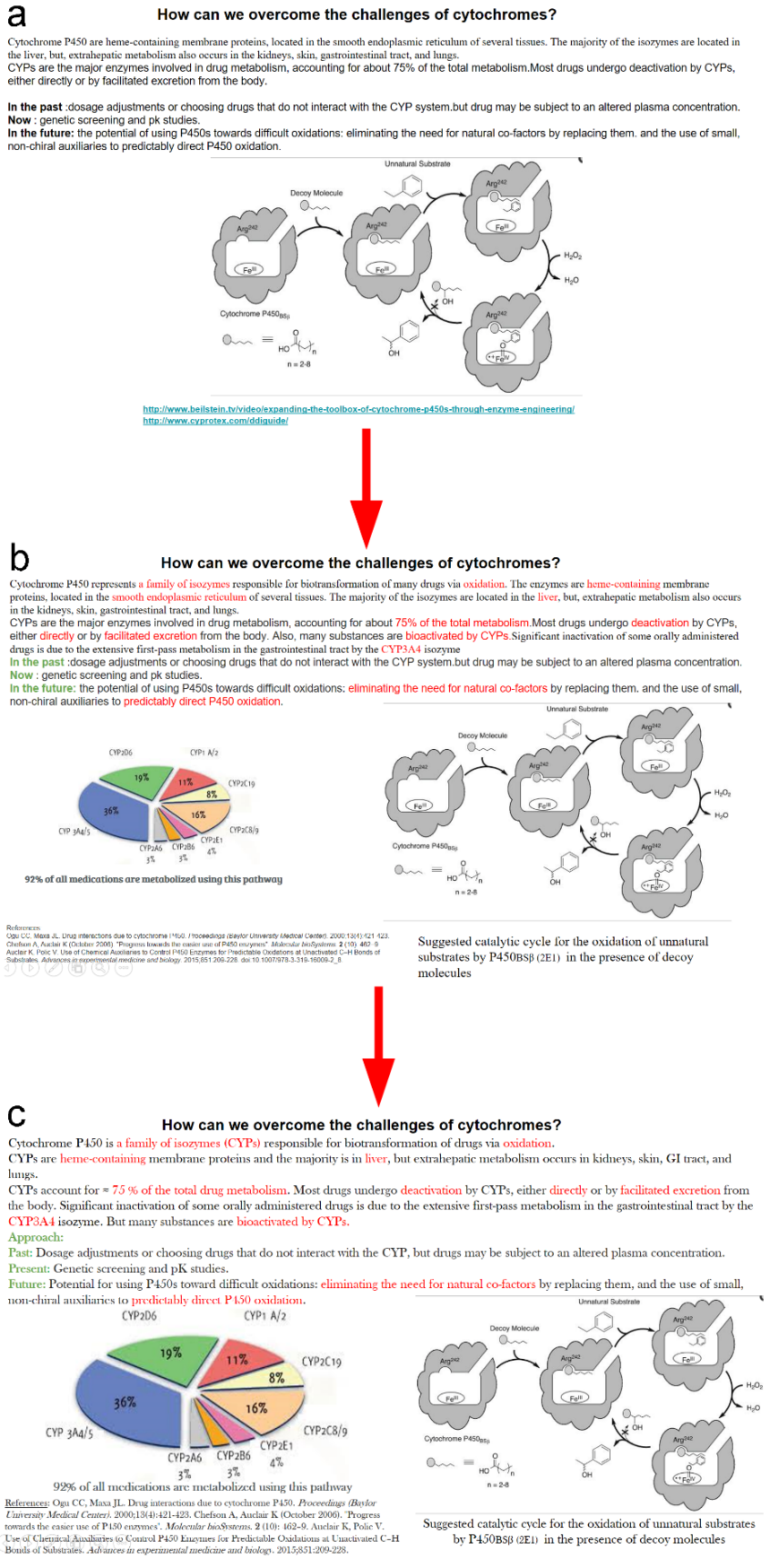


Fig.S1. Timeline of the evolution of the form of the co-created slide on the topic of overcoming the challenges of the cytochrome from after the homework stage, where the student worked alone on the content creation (a), to after its revision in response to the input received during the class (b), to the final adjustments for clarity after the second revision (c). The first round of revisions referred to correct referencing, additional details and a complementary figure. Other slides co-created in the class underwent a typically greater degree of change during the same timeline progression from (a) to (c).


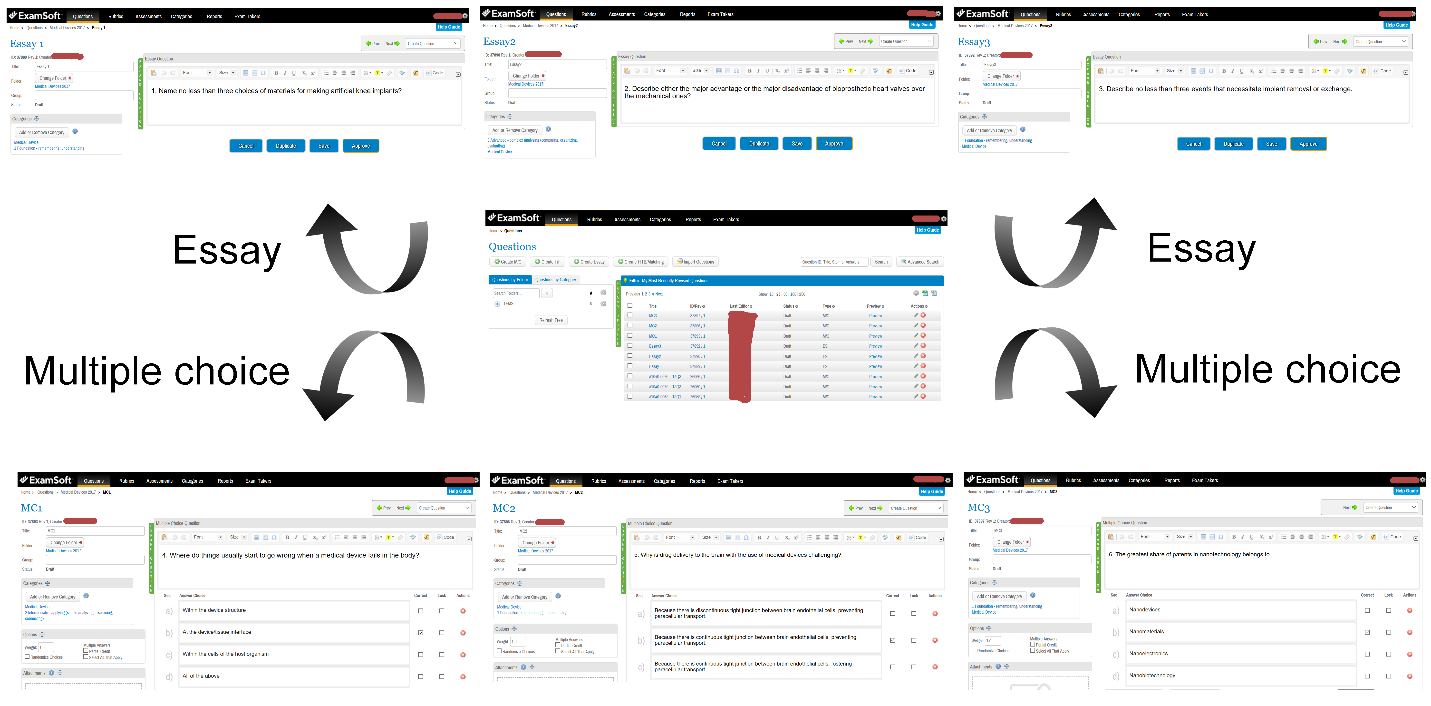


Fig.S2. Examples of test questions matched with the cognitive level categories and delivered using the ExamSoft platform.
